# Supplementary material for: MetaRibo-Seq measures translation in microbiomes
Source: Nat Commun. 2020 Jun 29;11:3268. doi: 10.1038/s41467-020-17081-z (PMC7324362; doi:10.1038/s41467-020-17081-z)
Supplement: Supplementary file 10 — Supplementary Data 7 [file 41467_2020_17081_MOESM10_ESM.zip › File2/Confidence_High_Taxonomy/379105_out.krona.html]

Javascript must be enabled to view this page.

members
magnitude
magnitudeUnassigned
count
unassigned
taxon
rank

379105\_out

13

13
2
superkingdom

13
phylum
1239

12
class
186801

order
186802
12

1
family
186803

genus
841
1

species
166486
1

SRS148721\_contig\_number\_55204

11
31979
family

11
1485
genus

59620
species

SRS015065\_contig\_number\_contig-100\_36196.77692SRS015854\_contig\_number\_5725SRS019068\_contig\_number\_102672SRS046717\_contig\_number\_contig-100\_20300.20301SRS058723\_contig\_number\_contig-100\_2371.36139SRS076929\_contig\_number\_25074SRS098073\_contig\_number\_17364SRS098644\_contig\_number\_contig-100\_944.194618SRS103987\_contig\_number\_contig-100\_31115.72815SRS144506\_contig\_number\_5689SRS148159\_contig\_number\_44098
11

1
526524
class

526525
order
1

family
128827
1

genus
100883
1

species
2293076
1

SRS016095\_contig\_number\_14766
